# Circulars of Information of Bureau of Education

**Published:** 1873-12

**Authors:** 


*Circulars of Information of the Bureau of Education*, Washington D. C.. Nos. 1, 2, 3, and 4 for 1873, relating to College Graduates, College Students, vital statistics in the U. S., Schools in British India, College commencements of 1873, and List of Publications by members of College Faculties and Learned Societies in the U. S. 1867—1872. Printed at the Government Printing Office.

